# Method of Glyphosate, AMPA, and Glufosinate Ammonium Determination in Beebread by Liquid Chromatography—Tandem Mass Spectrometry after Molecularly Imprinted Solid-Phase Extraction

**DOI:** 10.3390/molecules27175741

**Published:** 2022-09-05

**Authors:** Marta Małysiak, Tomasz Kiljanek

**Affiliations:** Department of Pharmacology and Toxicology, National Veterinary Research Institute, Aleja Partyzantów 57, 24-100 Puławy, Poland

**Keywords:** glyphosate, beebread, bees, pesticide residue analysis, molecularly imprinted polymers, derivatization, LC-MS/MS

## Abstract

The aim of this study was to develop a method for the determination of glyphosate, its metabolite aminomethylphosphonic acid (AMPA), and glufosinate ammonium residues in beebread samples, which could then be used to assess bees’ exposure to their residues. The complexity of beebread’s matrix, combined with the specific properties of glyphosate itself, required careful selection and optimization of each analysis step. The use of molecularly imprinted solid-phase extraction (MIP-SPE) by AFFINIMIP glyphosate as an initial clean-up step significantly eliminated matrix components and ensured an efficient derivatization step. Colorless beebread extracts were derivatized by the addition of 9-fluorenylmethyl chloroformate (FMOC-Cl). After derivatization, in order to remove FMOC-OH and residual borate buffer, a solid-phase extraction (SPE) clean-up step using Oasis HLB was carried out. Instrumental analysis was performed by liquid chromatography coupled with tandem mass spectrometry (LC–MS/MS). The method was validated according to the SANTE/11312/2021 guideline at concentrations of 5, 10, and 100 µg/kg, and satisfactory recovery (trueness) values (76–111%) and precision (RSDr) ≤ 18% were obtained. The limit of quantification (LOQ) was 5 µg/kg for AMPA and glufosinate ammonium and 10 µg/kg for glyphosate. The method was positively verified by the international proficiency test. Analysis of beebread samples showed the method’s usefulness in practice. The developed method could be a reliable tool for the assessment of beebread’s contamination with residues of glyphosate, its metabolite AMPA, and glufosinate ammonium.

## 1. Introduction

Glyphosate is a non-selective herbicide, and is one of the most used pesticides in the world. Glyphosate-based herbicides used for the elimination of flowering weeds reduce the availability of forage and the diversity of food resources for bees. Bees could have direct contact with glyphosate during its application on flowering weeds or indirect contact through contaminated pollen and nectar. Glyphosate by itself is only mildly toxic to bees, and its median lethal dose (LD_50_) is above 100 µg per bee [1]. This low toxicity enables the transfer of glyphosate to the hive, where it may present risks associated with long-term exposure. Glyphosate has been identified as one of the pesticides of greatest concern in terms of sublethal effects on bees [2]. Glyphosate acts on the same enzyme as occurs in the bees gut symbionts, consequently leading to weakened microflora and increased susceptibility to infection by pathogens, potentially increasing their mortality [3,4]. Chronic exposure of honeybee larvae to glyphosate leads to increased catabolism and oxidative metabolism, and may consequently cause long-term negative effects on bee populations [5]. A recent study showed that long-term glyphosate exposure impairs collective thermoregulation in bumblebees [6].

Beebread is a food for honeybee larvae. It consists of a fermented mixture of pollen, honey or nectar, and secretions of bees’ salivary glands. Beebread has a rich composition of proteins, carbohydrates, lipids, vitamins, phytosterols, fatty acids, and phenolic compounds. 

Beebread is a possible source of long-term exposure of bees to glyphosate. However, only a few studies on glyphosate residues in beebread have been published. Glyphosate was identified in 50% of beebread samples in Italy using the Quick Polar Pesticides (QuPPe) direct method [7]. In a Belgian study, glyphosate was identified in 81.5% of beebread samples analyzed by an indirect method with simple clean-up using a C18 solid-phase extraction (SPE) and 9-fluorenylmethyl chloroformate (FMOC-Cl) derivatization [8]. The development of a reliable and sensitive method of glyphosate determination in beebread is crucial to assess long-term exposure and to further study its sublethal effects on bees.

Glyphosate, its metabolite aminomethylphosphonic acid (AMPA), and glufosinate ammonium, due to their low molecular weight, zwitterionic nature, and high polarity, cannot be analyzed with other pesticides by multiresidue methods. These herbicides require specific analytical conditions and single-residue methods. Currently, two types of methods are used: direct methods, or those involving a derivatization step. Direct methods are quick due to their very limited sample preparation step—extraction by water/methanol [9], methanol/1% formic acid [10], or water with the addition of NH4-COOH/HCOOH as a buffering agent [11]. An optional clean-up step may be applied as SPE with C18 sorbent [12], Oasis HLB [13], ion-exchange Bond Elut Plexa PAX [14], InertSep SAX, or Oasis MCX [15]. However, due to the highly polar properties of glyphosate, glufosinate, and AMPA, these compounds cannot be directly separated by reverse-phase chromatography. Ion chromatography with an anion-exchange analytical column would provide better retention of analytes with a strongly anionic phosphonate structure [15]. Polar herbicides may also be determined by hydrophilic interaction chromatography (HILIC) [16,17] or by porous graphitic carbon chromatography with a Hypercarb column [12,14].

Among the indirect methods, three types of glyphosate derivatization steps are described in the literature: Pre-column derivatization with the use of FMOC-Cl reagent reduces the polar character of glyphosate, thereby facilitating chromatographic retention, and enabling analysis by liquid chromatography with mass spectrometry [18,19,20]. Post-column derivatization, based on the conversion of glyphosate into glycine and derivatization with o-phthalaldehyde, results in a product detected by a fluorescent detector [21,22,23]. Volatile and stable molecules necessary for gas chromatography determinations are obtained using trifluoroacetic acid anhydride and heptafluorobutanol in the derivatization step of glyphosate and AMPA [24,25].

Developing a sensitive method for determining glyphosate in beebread is extremely difficult. Beebread contains a wide variety of matrix components that particularly interfere in the analysis of glyphosate. Matrix components in the final extract cause strong suppression of the analyzed ions, causing extreme underestimation of the sensitivity of analytical methods. In turn, insufficient purification of extracts results in rapid contamination of the mass spectrometer. The introduction of an effective clean-up step in the analysis of glyphosate in beebread is essential, but is also extremely difficult due to the very specific properties of this substance—its polarity and amphoterism, which significantly limit the range of possible purification methods. The use of specially synthetized molecularly imprinted polymers (MIPs) to clean up glyphosate residues from matrix components seems extremely promising. In recent years, MIPs with tailor-made recognition sites have been used for the analysis of organic pollutants in water and food samples [26,27]. MIPs reduce interferences and matrix effects during sample preparation, and result in selective and precise analytical methods by enhancing chromatographic separation and detection. MIPs’ exceptional efficiency has also been utilized in the analysis of glyphosate residues in water samples [28,29]. However, MIPs have not yet been tested in complex matrices such as beebread.

The aim of this study was to develop a sensitive and reliable method specifically for the determination of glyphosate, AMPA, and glufosinate ammonium residues in the complex beebread matrix. To the best of our knowledge, this would be the first fully optimized, validated, and verified method of glyphosate determination in beebread. The use of this method in practice will provide most reliable data on the possible exposure of bees to residues of glyphosate.

## 2. Results and Discussion

### 2.1. Method Development

**Figure 1:**
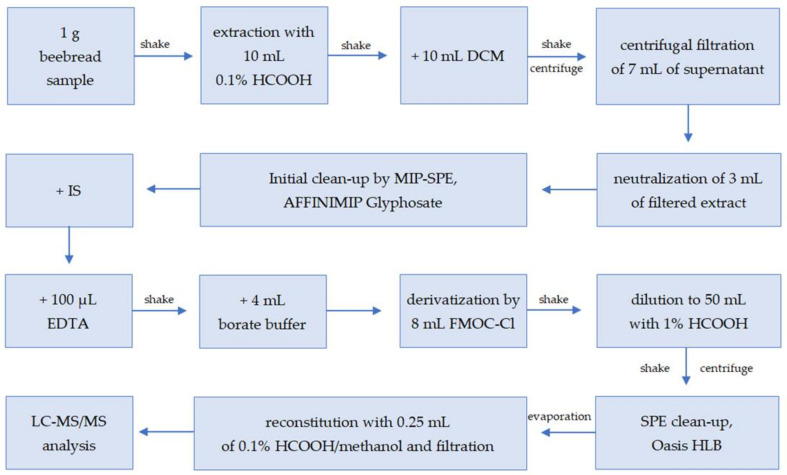
Scheme of sample preparation of beebread samples for glyphosate, AMPA, and glufosinate ammonium analysis (DCM: dichloromethane, MIP-SPE: molecularly imprinted solid-phase extraction, IS: internal standard, EDTA: ethylenediaminetetraacetic acid disodium salt dehydrate, LC—MS/MS: liquid chromatography coupled with tandem mass spectrometry).

In the first stage, the possibility of analyzing glyphosate in beebread was tested using the Quick Polar Pesticides (QuPPe) direct method of chromatographic analysis on a Hypercarb column [12]. The QuPPe method enabled the analysis of beebread, and its significant advantage was the simplicity and rapidity of sample preparation; however, this also resulted in insufficient purification of this very complex matrix, as well as its inability to be used in routine analysis due to the hazard of significant contamination of the mass spectrometer. Thus, a sample preparation procedure for the determination of glyphosate, glufosinate ammonium, and their metabolites through an FMOC derivatization step [19] was selected as the starting point for the development of a method suitable for the beebread matrix. The sample preparation procedure for glyphosate residue analysis was developed with careful selection and optimization of each step. The final sample preparation procedure is described in Section 3.3 of the Materials and Methods and shown in Figure 1.

Glyphosate in a derivative-free state can bind to active sites on glass; therefore, polypropylene materials were used to avoid analyte losses.

#### 2.1.1. Extraction Step

The sample size was set to 1 g. This value was chosen to enable the smallest possible sample while ensuring the highest possible sensitivity of the method. The quantity of beebread is often very limited in practice, especially in the case of solitary bees or bumblebees. During field studies, the analysis of glyphosate residues complements multiresidue pesticide analysis or pollen origin analysis, which require additional samples. The use of 1 g test portions reduces the presence of interfering matrix components, leading to easier purification. Sample dilution is also a strategy used in the analysis of glyphosate residues, especially in view of the considerable problems in isolating it from the sample matrix. However, this approach requires the use of equipment with the highest sensitivity [30].

Acidified water (0.1% HCOOH) was chosen for the extraction of analytes from the beebread samples. The acidic extraction environment promoted the decomposition of the eventual analyte complexes with metals, increasing the accessibility of glyphosate and AMPA to FMOC.

#### 2.1.2. Derivatization Step

Before derivatization with FMOC-Cl, two steps were added: First ethylenediaminetetraacetic acid disodium salt dehydrate (EDTA) was added as a complexing agent to prevent metal interferences at alkaline pH [31]. Then, borate buffer was added to the extract in order to maintain an alkaline environment and ensure the stability of the derivatization reaction. The addition of 4 mL of borate buffer to the extract provided a pH value of 8.0. Derivatization reactions are often carried out in samples with a pH value of 9 [32,33,34]. Insufficient sodium tetraborate may result in low buffering capacity and, thus, low derivatization efficiency. It was checked whether the addition of more borate buffer (6 mL) and, thus, a higher pH (pH = 9.0), would result in better derivatization efficiency. The results of this study show that increasing the amount of buffer has an insignificant influence on recovery.

**Figure 2:**
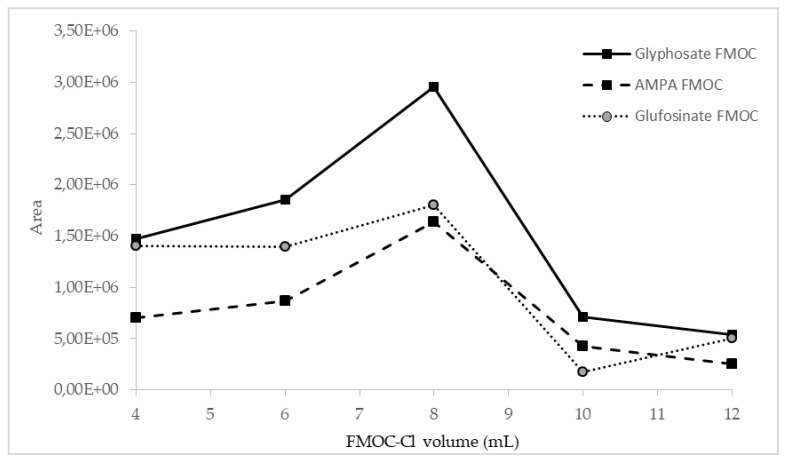
Influence of FMOC-Cl volume on derivatization efficiency, observed as peak areas of analyte derivatives.

The influence of the amount of FMOC-Cl on the efficiency of the derivatization process was investigated. Preliminary studies showed that the addition of 4 mL of 20 mM FMOC-Cl may be insufficient, so further investigation was performed with the addition of 6, 8, 10, and 12 mL of FMOC-Cl. The best results for all compounds were obtained in the case of derivatization with the use of 8 mL of 20 mM FMOC-Cl, as shown in Figure 2.

The derivatization process was also carried out with a smaller volume of more concentrated FMOC-Cl (4 mL, 40 mM), but this resulted in a ninefold signal reduction for AMPA and a sixfold signal reduction for glyphosate. This shows that 8 mL of 20 mM FMOC-Cl in acetonitrile is the most optimal formulation for derivatization efficiency.

#### 2.1.3. Post-Derivatization Clean-Up Step

The main problem of derivatization with FMOC-Cl is the formation of FMOC-OH, which is less soluble in water than analyte derivatives. The FMOC-OH is formed as a result of the hydrolysis and decarboxylation reaction of excess FMOC-Cl. It can precipitate in the chromatographic column or ion source and decrease the ionization efficiency, reducing the sensitivity of the mass spectrometer. Moreover, previous studies showed that an excess of borate buffer after derivatization can decompose the glyphosate-FMOC derivative [32].

A post-derivatization SPE clean-up step with Oasis HLB was introduced in order to eliminate any remaining FMOC-OH and borate buffer. Before loading the extract onto the SPE cartridge, the sample was diluted. The addition of an appropriate volume of concentrated formic acid, corresponding to the quantity of acid in the diluted sample, caused twofold lower recoveries for AMPA and glufosinate, and fourfold lower recovery for glyphosate. 

Initially, the extract loaded onto the Oasis HLB SPE cartridge was washed with dichloromethane (DCM) before the elution of glyphosate with methanol. However, traces of DCM were still present in the eluate, and extended the time of its evaporation. Thus, ethyl acetate was tested in order to remove the remaining DCM from the Oasis HLB cartridge. The recoveries of AMPA and glyphosate increased with the use of ethyl acetate, and the time of evaporation was shortened.

The drawback of the post-derivatization clean-up step is the high volume of the extract loaded on the Oasis HLB cartridge, which extends the time necessary for this clean-up step. Attempts have been made to reduce the volume of the extract by the addition of an appropriate volume of more concentrated formic acid, corresponding to the same quantity of formic acid. However this attempt caused twofold lower recoveries for AMPA and glufosinate ammonium, and fourfold lower recovery for glyphosate. The extract must be diluted to 50 mL with 1% formic acid in order to improve clean-up by the Oasis HLB cartridge.

#### 2.1.4. Initial Clean-Up Step

The introduction of an additional clean-up step prior to derivatization was necessary, taking into account the complexity of the beebread matrix. The matrix components still caused strong suppression of the analyzed ions, even though Oasis HLB SPE clean-up was performed after derivatization. 

First, DCM was added to the extract in order to remove hydrophobic matrix components. Due to the polar nature of the tested compounds, they are insoluble in DCM [19].

**Figure 3:**
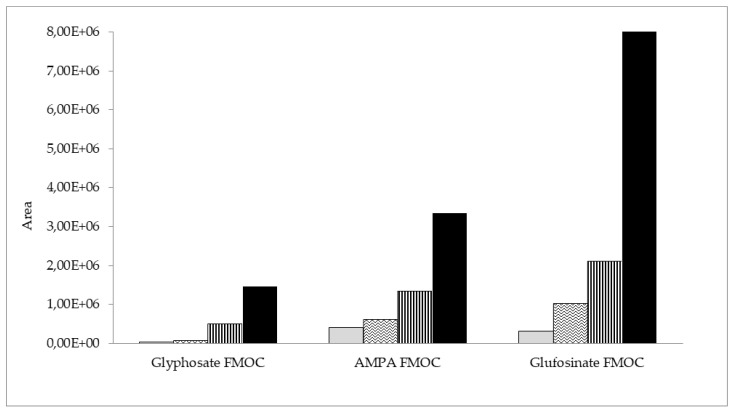
Peak areas of AMPA, glyphosate, and glufosinate FMOC derivatives in beebread samples initially cleaned up via four different methods: dSPE with C18 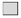, SPE with C18 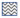, SPE with Strata X 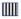, and MIP-SPE by AFFINIMIP glyphosate 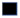.

Furthermore, four initial clean-up methods were compared: dispersive solid-phase extraction (dSPE) with C18 sorbent (500 mg), SPE with C18 (500 mg), Strata X (200 mg), and molecularly imprinted solid-phase extraction (MIP-SPE) with an AFFINIMIP glyphosate cartridge. The results of the initial clean-up step tests with beebread samples spiked with AMPA, glyphosate, and glufosinate ammonium are shown in Figure 3. The best results and the highest peak areas for the analyte derivatives were obtained in the case of AFFINIMIP glyphosate MIP-SPE clean-up. The lowest detector signal was obtained for dSPE with C18 sorbent. SPE with the use of C18 and Strata X showed much worse results than AFFINIMIP glyphosate. The extract obtained after clean-up with the MIP-SPE method was clear and almost colorless, and this approach was ultimately selected.

Commercially available AFFINIMIP glyphosate cartridges have previously been used during glyphosate analysis in water samples [35,36] and wine samples [37]. The present study is the first application of AFFINIMIP glyphosate for complex beebread samples.

The method of purification of extracts using MIP-SPE differs significantly from methods with C18 or Strata X sorbents, which adsorb unwanted matrix components. In the case of MIP-SPE by AFFINIMIP glyphosate, the molecules build into a crosslinked selective polymer, whilst matrix components are washed out from the SPE cartridge with water. AFFINIMIP glyphosate, due to its selective recognition properties for the target glyphosate molecules, works by means of a “lock and key” mechanism [27].

In the next step, glyphosate was eluted using 0.1 M HCl. The MIP-SPE approach allows better availability of glyphosate molecules at the subsequent derivatization step, along with a significantly reduced ion suppression effect. Moreover, the low pH value of the eluate (pH = 1) obtained after MIP-SPE increases the protonation of glyphosate, AMPA, and glufosinate ammonium, protecting the analytes against eventual complexation. Previous studies showed that the use of a pH value of 1 significantly improves the recovery of glyphosate [38].

Extract cleaned up with an AFFINIMIP cartridge should have a pH value within the range of 6 to 9, according to the producer’s declaration. The pH value of the beebread extract therefore had to be adjusted, because it was around 3. The impact of four different pH values of beebread extract on MIP-SPE clean-up efficiency was compared: no pH value adjustment, and adjustment of pH to 5.5, 7.0, and 9.0. The adjustment of the beebread extract’s pH value to 9 caused the worst glyphosate recovery of all cases tested. At a pH value of 3, significantly (about sevenfold) lower recoveries were obtained for AMPA, and the recoveries for glyphosate and glufosinate ammonium also decreased. For a pH of 5.5, the recoveries were similar to the results obtained when pH was adjusted to 7.0; however, the glyphosate response was better at a pH value of 7.0. Thus, 3 mL of filtered beebread supernatant was neutralized to a pH value of 7.0 by the addition of NH_4_OH; this was chosen as the optimal pH value of beebread extract before the initial MIP-SPE clean-up step.

### 2.2. Method Validation and Verification

The results of the validation process met the criteria of SANTE/11312/2021 [39]. The average recovery, as a measure of accuracy, ranged between 76 and 111%, and the relative standard deviation (RSDr), representing the precision, was ≤18%. The limit of quantification (LOQ) for glufosinate ammonium and AMPA was established as 5 µg/kg, and for glyphosate was 10 µg/kg. The developed method showed good linearity within the range from the LOQ to 500 µg/kg, with correlation coefficient (R^2^) values above 0.98. All back-calculated concentrations of calibration points were within ±20% of their nominal values. Significant suppression of ionization in the presence of the matrix was demonstrated for all three determined compounds, with a matrix effect (ME) below -50%. Procedural standard calibration was used in order to compensate for the matrix effect. Detailed validation results are presented in Table 1.

The method was satisfactory, as verified by the international proficiency test “Progetto Trieste–Pesticide Residues 2020, Round of October” organized by Test Veritas S.r.l, Laboratory Proficiency Testing for Food Analysis. The developed method was used to analyze glyphosate residues in honey, and achieved a satisfactory *z*-score value of 0.03.

### 2.3. Real Sample Application

Analysis of eight beebread samples from online sales revealed the presence of glyphosate residues in seven of them. Glyphosate was found in concentrations as high as 320 µg/kg, and the median result was 96 µg/kg. No residues of AMPA or glufosinate ammonium were determined in any of the beebread samples tested. The obtained results of preliminary analyses confirm the possibility of contamination of beebread with glyphosate residues, and are consistent with the results obtained in Belgium [8]. In all probability, glyphosate does not kill flowering plants immediately after its application, and bees can collect contaminated pollen from sprayed flowers for some period of time. These results indicate the need for further research on the contamination of beebread with glyphosate residues, the purpose of which should be both to assess the exposure of bees and to evaluate the risks associated with it.

## 3. Materials and Methods

### 3.1. Chemicals and Reagents

Aminomethylphosphonic acid (AMPA, 99.9% purity), aminomethylphosphonic acid 13C 15N (AMPA ^13^C ^15^N; 100 µg/mL) (99.0%), glyphosate—FMOC (95.8%), glufosinate ammonium—FMOC (96.2%), and AMPA—FMOC (99.6%) were purchased from Dr. Ehrenstorfer (Augsburg, Germany). Glyphosate (99.7%), glufosinate ammonium (99.6%), and glyphosate-2-13C15N (99.6%) were from Sigma-Aldrich (Buchs, Switzerland). HPLC-grade DCM, ultra-LC—MS-grade methanol, LC—MS-grade acetonitrile, and Ultra-Resi-Analyzed ethyl acetate were obtained from the J.T. Baker brand of Avantor Performance Materials (Deventer, The Netherlands). EDTA, FMOC-Cl (97%), and sodium tetraborate decahydrate of ACS grade were purchased from Sigma-Aldrich (Buchs, Switzerland). Ammonia solution (25%) and 0.1 M hydrochloric acid were supplied by the POCH brand of Avantor Performance Materials (Gliwice, Poland). Formic acid eluent additive for LC—MS was purchased from Honeywell (Seelze, Germany). SPE Oasis HLB cartridges (200 mg, 6 mL) were purchased from Waters (Massachusetts, USA). AFFINIMIP SPE glyphosate cartridges (6 mL) were purchased from AFFINISEP (Le Houlme, Normandy, France). Deionized water was obtained using a Milli-Q Plus system from Merck (Billerica, Massachusetts, USA). PVDF nonsterile centrifugal filters (0.22 µm, 25 mL) were purchased from Thermo Fisher Scientific (Waltham, Massachusetts, USA), and a Nanosep® centrifugal device with a Bio-Inert^®^ 0.2 µm membrane was obtained from Pall Corporation (Puerto Rico, USA).

### 3.2. Standard Solutions

Individual stock standard solutions at concentrations of 1000–2400 µg/mL were prepared in 10% acetonitrile in water solution and stored in plastic (glyphosate and AMPA) or glass (glufosinate ammonium) containers at a temperature below −18 °C. Mixed standard solutions for validation and calibration were prepared using appropriate dilutions of stock standard solutions with deionized water.

Mixtures of internal standards at concentrations of 10 µg/mL for glyphosate-2-^13^C^15^N and 1 µg/mL for AMPA^13^C^15^N were prepared in deionized water.

### 3.3. Sample Preparation

A 1 g sample of homogenized beebread was weighed into a 50 mL centrifuge tube. Then, 10 mL of 0.1% formic acid in aqueous extraction solution was added, and the sample was shaken in a MiniG mechanical disrupter with a vertically moving platform (SPEX Sample Prep, Metuchen, NJ, USA) for 2 min. Next, 10 mL of dichloromethane was added, and the sample was shaken again for 3 min. At this point, the sample was centrifuged at room temperature and RCF = 3850× *g* for 10 min. Then, 7 mL of supernatant was transferred into a centrifugal PVDF filter with 0.22 µm pore diameter, and it was centrifuged at RCF = 2500× *g* for 7 min. After that, 3 mL of extract was transferred to another plastic tube, where its pH value was neutralized by the addition of ammonia solution. 

The sample was transferred onto an AFFINIMIP glyphosate SPE cartridge, previously conditioned with 6 mL of deionized water, and washed with 12 mL of deionized water. The analytes were eluted with 8 mL of 0.1 M hydrochloric acid into a plastic tube. Then, 50 µL of the internal standard mixture solution was added, and 100 µL of 0.4 M EDTA in aqueous solution was added to the sample and shaken again. 

The extract was transferred into a 50 mL centrifuge tube and mixed with 4 mL of 5% sodium tetraborate decahydrate in aqueous solution as a buffering agent. For the derivatization step, 8 mL of 20 mM FMOC-Cl in acetonitrile solution was added, and the sample was vortexed with the BenchMixer XL Multi-Tube Vortexer (Benchmark Scientific, Edison, NJ, USA) for 5 min and left overnight at room temperature. Then, the extract was filled to a volume of 50 mL with 1% formic acid, shaken, and centrifuged at RCF = 3850× *g* for 10 min. The supernatant was transferred into another tube to eliminate the sediment. 

The extract was loaded onto the Oasis HLB SPE cartridges, which had already been conditioned with 10 mL of methanol and 10 mL of 1% formic acid in water. After loading, the cartridges were washed with 10 mL of deionized water and 10 mL of dichloromethane and dried under a vacuum. Then, the cartridges were washed with 2 mL of ethyl acetate and dried again under the vacuum. The analytes were eluted via SPE with 5 mL of methanol. The eluate was evaporated to dryness under a gentle stream of nitrogen and reconstituted with 0.25 mL of a mixture consisting of 0.1% formic acid in water and methanol at a ratio of 1:1(*v*/*v*). The sample was filtered using the Nanosep® centrifugal device with a Bio-Inert® 0.2 µm membrane (RCF = 14,000× *g*, 30 s) and transferred into a plastic vial for liquid chromatography coupled with tandem mass spectrometry (LC—MS/MS) analysis.

### 3.4. LC—MS/MS Analysis

The chromatographic analysis was performed on a Shimadzu LC-20AD XR equipped with a CBM-20A system controller, LC-20AD XR pump, SIL-20AC XR autosampler, CTO-30A column oven, and DGU-20A5R degasser. The injection volume was 20 µL. Chromatographic separation was achieved using a Zorbax Eclipse Plus C18 column (2.1 × 150 mm, 3.5 µm) operated at 35 °C. The mobile phase that was applied consisted of methanol as phase A and 5 mM ammonium formate in pH 8 water as phase B. Separation was performed at a flow rate of 0.4 mL/min, with a gradient elution starting at 90% phase B initially held for 0.5 min, decreased to 10% for 6.5 min and held for 2 min, and then increased to 90% over 1 min and held constant until the end of analysis. The total time of analysis was 20 min.

As a tandem mass spectrometer, the QTRAP 5500 from SCIEX (Framingham, MA, USA) with a Turbo V Source was used. Ionization by ESI in negative mode (−4500 V) was achieved with an ion source at 500 °C. The ion source gases (i.e., curtain gas, nebulizer gas, and heater gas) were nitrogen at 35, 50, and 50 psi, respectively. Optimized precursor ion–product ion MRM transitions and corresponding values of LC—MS/MS parameters (i.e., declustering potential (DP), collision energy (CE), and collision cell exit potential (CXP)) for each compound are shown in Table 2. Quantitative analysis was based on MRM 1 transition, whilst MRM 2 was used for qualitative analysis. Chromatograms of glyphosate—FMOC, AMPA—FMOC, and glufosinate ammonium—FMOC derivatives for quantitative and qualitative analysis are shown in Figures S1–S3 in the Supplementary Materials. Instrument control and data acquisition were carried out using Analyst 1.7 software with a scheduled MRM advanced mode. Quantitative and qualitative analyses were performed using MultiQuant software version 3.0.

### 3.5. Validation and Verification

Our method was validated according to SANTE/11312/2021 [39]. The linear range of the method was determined by two replicate analyses of beebread samples, spiked at levels 5, 10, 50, 100, and 500 µg/kg with analytical standard solutions of glyphosate, AMPA, and glufosinate ammonium employing stable isotope-labeled internal standards (procedural calibration). For glyphosate and AMPA, the corresponding internal standards were used, while for glufosinate ammonium the same internal standard as for glyphosate was used.

The LOQ is the lowest concentration of a compound that can be quantified with acceptable accuracy and precision. Analysis of the beebread samples spiked with glyphosate, AMPA, and glufosinate ammonium at the levels of 5, 10, and 100 µg/kg, with five replications for each level, was carried out to evaluate accuracy and precision. The matrix effect was established by comparison of the slope received for the procedural standard calibration curve and the solvent calibration curve.

The performance of the method and the correctness of the selection and optimization of each step of analysis were verified using the international proficiency test.

### 3.6. Real Application of Samples 

The developed and validated method was used in the analysis of commercially available beebread samples.

## 4. Conclusions

The developed method allows the determination of glyphosate, AMPA, and glufosinate ammonium in beebread matrix. To the best of our knowledge, this is the first published method for the determination of glyphosate specifically dedicated to the analysis of beebread. Each step of the method was optimized to enable the most sensitive and reliable analysis of glyphosate, AMPA, and glufosinate ammonium residues in beebread. The MIP-SPE technique used as an initial clean-up step resulted in a substantial cleaning of the matrix components, and ensured high derivatization efficiency. Optimized derivatization conditions ensured high sensitivity of the method. Post-derivatization clean-up eliminated the possibility of mass spectrometer contamination. Further use of this method in practice will provide most reliable data on the exposure of bees to glyphosate residues—one of the most widely used pesticides in the world today.

## Figures and Tables

**Table 1:** Validation results of the developed method for the determination of glyphosate, AMPA, and glufosinate ammonium in beebread.

Compound	Linearity Range, µg/kg	R^2^	ME, %	Recovery, % (RSDr, %)		
				5 µg/kg	10 µg/kg	100 µg/kg
AMPA-FMOC	5–500	0.988	−86	111 (9)	97 (17)	76 (4)
Glyphosate-FMOC	10–500	0.998	−58	–	106 (18)	82 (5)
Glufosinate ammonium-FMOC	5–500	0.981	−90	76 (17)	105 (9)	76(5)

**Table 2:** MRM transitions and parameters of LC—MS/MS analysis for target compounds and internal standards (ISTD).

Compound	MRM 1	DP	CE	CXP	MRM 2	CE	CXP
Glyphosate–FMOC	390 > 150	−59	−37	−8	390 > 124	−39	−10
AMPA–FMOC	332 > 110	−55	−11	−7	332 > 136	−23	−8
Glufosinate ammonium–FMOC	402 > 180	−65	−16	−11	402 > 206	−22	−13
AMPA ^13^C ^15^N–FMOC (ISTD)	334 > 112	−37	−12	−1			
Glyphosate-2-^13^C^15^N–FMOC (ISTD)	393 > 171	−45	−18	−9			

## Data Availability

Not applicable.

## Supplementary Materials

The following supporting information can be downloaded at: www.mdpi.com/article/10.3390/molecules27175741/s1, Figure S1: Chromatograms of glyphosate—FMOC derivatives for quantitative (MRM 1, 390 > 150) and qualitative analysis (MRM 2, 390 > 124): (a) beebread sample spiked at the level of 100 µg/kg—quantitative; (b) beebread sample spiked at the level of 100 µg/kg—qualitative; (c) beebread sample spiked at the level of 10 µg/kg—quantitative; (d) beebread sample spiked at the level of 10 µg/kg—qualitative; (e) blank beebread sample—quantitative; (f) blank beebread sample—qualitative; Figure S2: Chromatograms of AMPA–FMOC derivatives for quantitative (MRM 1, 332 > 110) and qualitative analysis (MRM 2, 332 > 136): (a) beebread sample spiked at the level of 100 µg/kg—quantitative; (b) beebread sample spiked at the level of 100 µg/kg—qualitative; (c) beebread sample spiked at the level of 10 µg/kg—quantitative; (d) beebread sample spiked at the level of 10 µg/kg—qualitative; (e) blank beebread sample—quantitative; (f) blank beebread sample—qualitative; Figure S3: Chromatograms of glufosinate ammonium–FMOC derivatives for quantitative (MRM 1, 402 > 180) and qualitative analysis (MRM 2, 402 > 206): (a) beebread sample spiked at the level of 100 µg/kg—quantitative; (b) beebread sample spiked at the level of 100 µg/kg—qualitative; (c) beebread sample spiked at the level of 10 µg/kg—quantitative; (d) beebread sample spiked at the level of 10 µg/kg—qualitative; (e) blank beebread sample—quantitative; (f) blank beebread sample—qualitative.

## Sample Availability

Samples of the compounds are not available from the authors.
