# Supplementary material for: Method of Glyphosate, AMPA, and Glufosinate Ammonium Determination in Beebread by Liquid Chromatography—Tandem Mass Spectrometry after Molecularly Imprinted Solid-Phase Extraction
Source: Molecules. 2022 Sep 5;27(17):5741. doi: 10.3390/molecules27175741 (PMC9457744; doi:10.3390/molecules27175741)
Supplement: Supplementary file 1 [file molecules-27-05741-s001.zip › molecules-1874400-supplementary.pdf]

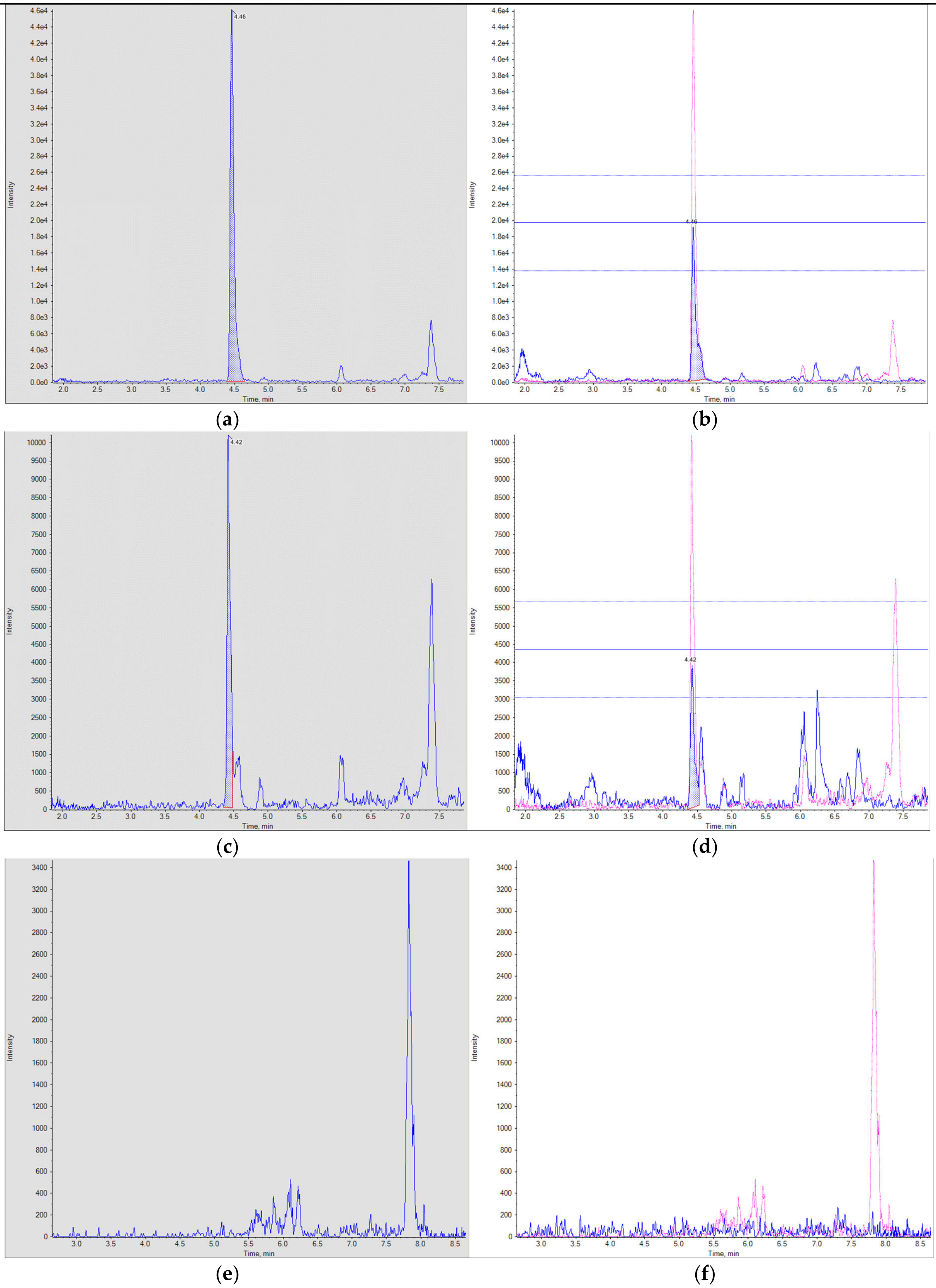

Figure S1. Chromatograms of Glyphosate-FMOC derivative for quantitative (MRM 1, 390>150) and qualitative analysis (MRM 2, 390>124): (**a**) beebread sample spiked at the level of 100 μg/kg – quantitative; (**b**) beebread sample spiked at the level of 100 μg/kg – qualitative; (**c**) Beebread sample spiked at the level of 10 μg/kg – quantitative; (**d**) Beebread sample spiked at the level of 10 μg/kg – qualitative; (**e**) Blank beebread sample – quantitative; (**f**) Blank beebread sample – qualitative.

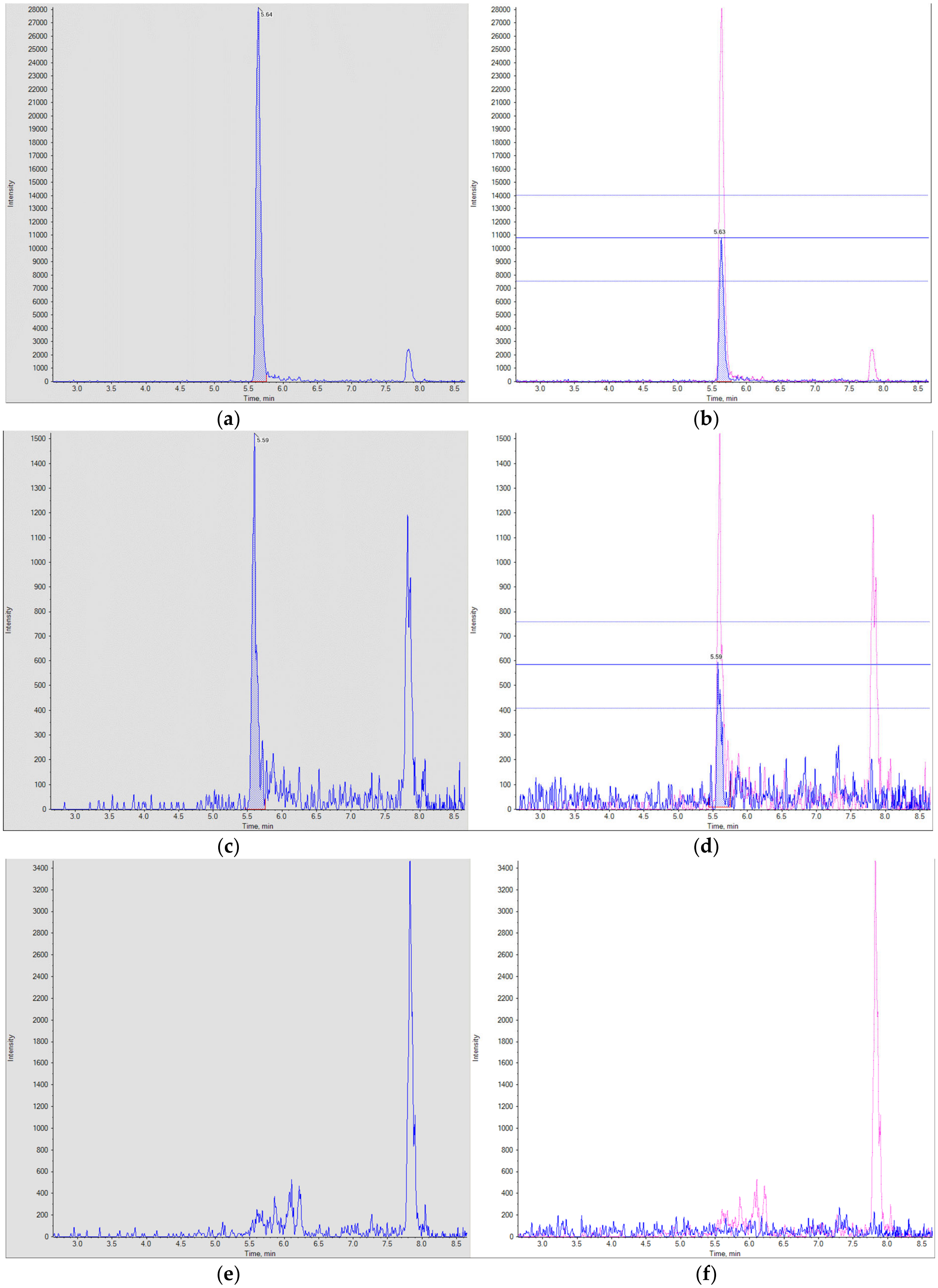

Figure S2. Chromatograms of AMPA-FMOC derivative for quantitative (MRM 1, 332>110) and qualitative analysis (MRM 2, 332>136): (**a**) beebread sample spiked at the level of 100 µg/kg – quantitative; (**b**) beebread sample spiked at the level of 100 µg/kg – qualitative; (**c**) Beebread sample spiked at the level of 10 µg/kg – quantitative; (**d**) Beebread sample spiked at the level of 10 µg/kg – qualitative; (**e**) Blank beebread sample – quantitative; (**f**) Blank beebread sample – qualitative.

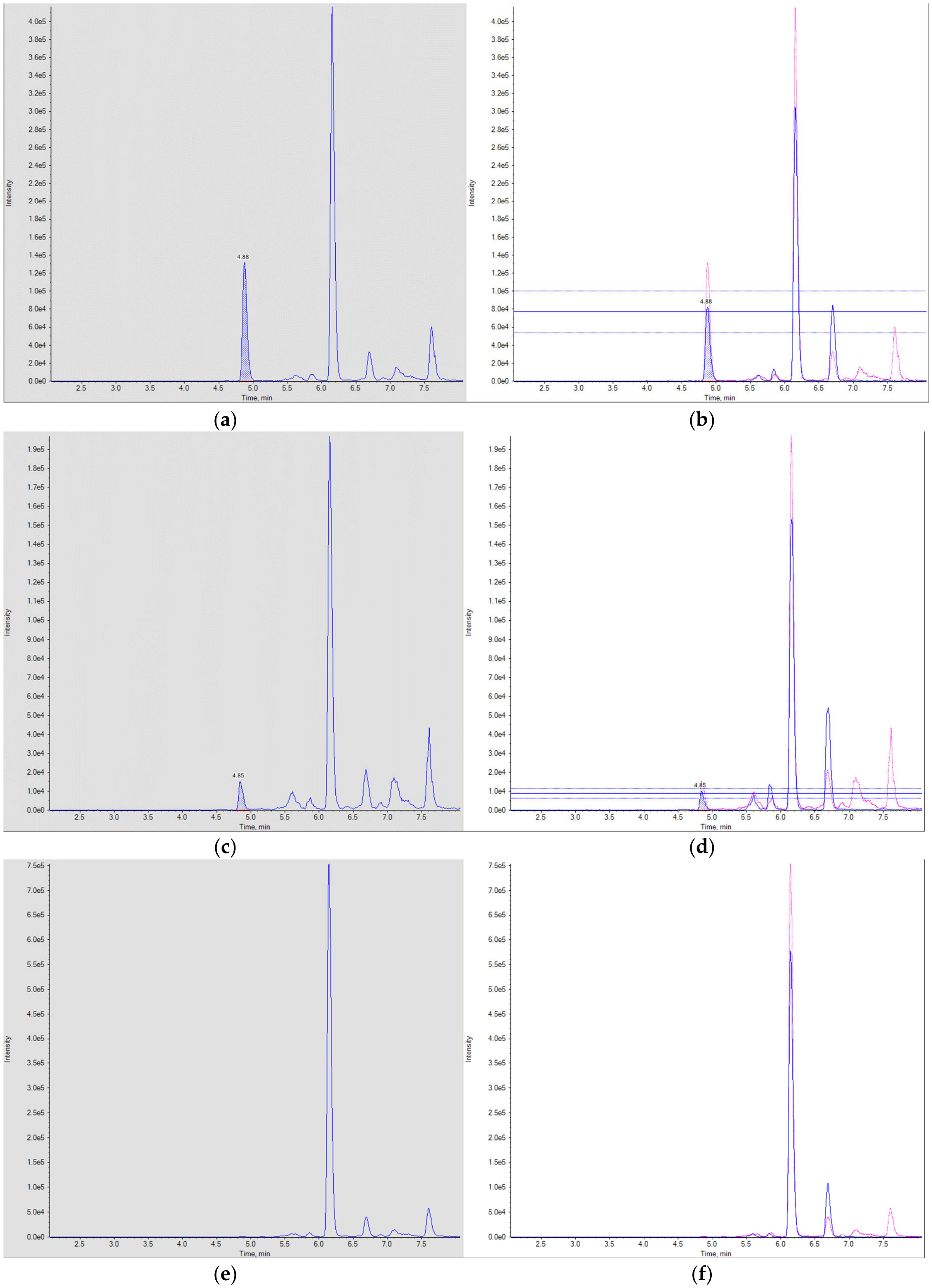

Figure S3. Chromatograms of Glufosinate ammonium-FMOC derivative for quantitative (MRM 1, 402>180) and qualitative analysis (MRM 2, 402>206): (**a**) beebread sample spiked at the level of 100 µg/kg – quantitative; (**b**) beebread sample spiked at the level of 100 µg/kg – qualitative; (**c**) Beebread sample spiked at the level of 10 µg/kg – quantitative; (**d**) Beebread sample spiked at the level of 10 µg/kg – qualitative; (**e**) Blank beebread sample – quantitative; (**f**) Blank beebread sample – qualitative.
